# Characterization of Postprandial Effects on CSF Metabolomics: A Pilot Study with Parallel Comparison to Plasma

**DOI:** 10.3390/metabo10050185

**Published:** 2020-05-06

**Authors:** Kosuke Saito, Kotaro Hattori, Tomohiro Andou, Yoshinori Satomi, Masamitsu Gotou, Hiroyuki Kobayashi, Shinsuke Hidese, Hiroshi Kunugi

**Affiliations:** 1 Division of Medical Safety Science, National Institute of Health Sciences, Kanagawa 210-9501, Japan; 2 Department of Mental Disorder Research, National Institute of Neuroscience, National Center of Neurology and Psychiatry, Tokyo 187-8502, Japan; hattori@ncnp.go.jp (K.H.); shidese@ncnp.go.jp (S.H.); hkunugi@ncnp.go.jp (H.K.); 3 Medical Genome Center, National Center of Neurology and Psychiatry, Tokyo 187-8551, Japan; 4 Axcelead Drug Discovery Partners, Inc., 26-1 Muraoka-Higashi 2-chome, Fujisawa, Kanagawa 251-0012, Japan; tomohiro.andou@axcelead.com; 5 Integrated Technology Research Laboratories, Pharmaceutical Research Division, Takeda Pharmaceutical Company Limited, 26-1 Muraoka-Higashi 2-chome, Fujisawa, Kanagawa 251-8555, Japan; yoshinori.satomi@shionogi.co.jp (Y.S.); ma.gotou@ono.co.jp (M.G.); hiroyuki.kobayashi@shionogi.co.jp (H.K.)

**Keywords:** CSF metabolites, metabolomics, lipidomics, postprandial effect

## Abstract

Cerebrospinal fluid (CSF) metabolites reflect biochemical diffusion/export from the brain and possibly serve as biomarkers related to brain disease severity, pathophysiology, and therapeutic efficacy/toxicity. Metabolomic studies using blood matrices have demonstrated interindividual and preanalytical variation of blood metabolites, whereas those of CSF metabolites remain unclear. In this study, we aimed to delineate the postprandial effects on CSF metabolites because fasting of patients with brain-related disorders is challenging. We collected pre- and postprandial (1.5, 3, and 6 h) plasma and CSF from nine healthy subjects. Using a mass-spectrometry-based global metabolomics approach, 150 and 130 hydrophilic metabolites and 263 and 340 lipids were detected in CSF and plasma, respectively. Principal component analysis of CSF hydrophilic metabolites and lipids primarily classified individual subjects at any time point, suggesting that the postprandial effects had a lower impact than interindividual variations on CSF metabolites. Individually, less than 10% of the CSF metabolites were putatively altered by postprandial effects (with either significant differences or over 2-fold changes, but not both) at any time point. Thus, global CSF metabolite levels are not directly associated with food intake, and except for several putatively altered CSF metabolites, postprandial effects are not a major concern when applying CSF metabolomics to screen biomarkers.

## 1. Introduction

Metabolomic approaches provide an overview of the profiles of metabolites such as amino acids, sugars, and lipids in biofluids and tissues [1,2,3]. Metabolites act as essential energy sources for cells, organs, and tissues, as well as key players for multiple biological processes. Their levels can reflect homeostatic alterations such as diseases and organ injuries. Metabolomics could thus be a useful tool for screening biomarkers of diseases and organ injuries and has already been applied in many diseases such as myocardial ischemia, hepatocellular carcinoma, and schizophrenia [4,5,6]. Currently, blood matrices such as plasma and serum are the most frequently used sources for screening biomarkers. Using blood matrices in metabolomics, interindividual variations such as age, gender, race, and BMI, as well as preanalytical variations such as sample preparation and fasting/postprandial status, have been well characterized. For example, androgens are male-enriched metabolites in Caucasians and Japanese [7,8], whereas sphingomyelins (SMs) are well-known female-enriched lipids [7,8,9,10]. In addition, sample preparation such as freezing and thawing affects the levels of biliverdin, bilirubin, and a broad spectrum of lipids [7,10], while postdrawing time to the preparation of plasma and serum alters the levels of cysteine, glutamic acid, arginine, ornithine, and phosphatidylserine (PS[38:4]) [11,12]. Postprandial effects on plasma metabolites have also been characterized in several reports [13,14,15]. Therefore, interindividual and preanalytical variations are major concerns in biomarker screening.

Cerebrospinal fluid (CSF), the biological fluid in the closest contact with the brain, is more specifically applied to search for biomarkers of brain diseases because CSF contains molecules of neural cell origin and can reflect the state of brain health and activity [16,17]. Some CSF biomarkers for brain disorders have already been established. For example, tau, phosphorylated tau, and Abeta42 are known biomarkers of Alzheimer’s disease and can be used to diagnose the disease with a high (~90%) sensitivity and specificity [18]. CSF metabolomics analyses have also demonstrated putative biomarkers for amyotrophic lateral sclerosis, Alzheimer’s disease, and Parkinson’s disease [19,20,21,22]. However, unlike metabolomics using blood matrices, the interindividual and preanalytical variations in CSF metabolomics remain unclear. In terms of the clinical application of CSF metabolomics, fasting treatment for patients with brain-related disorders such as schizophrenia and Alzheimer’s disease is a major ethical and practical concern. Therefore, determining the postprandial effects on CSF metabolites could provide valuable fundamental information for biomarker research using CSF metabolomics.

In this study, we recruited nine healthy subjects and collected both preprandial and postprandial plasma and CSF. The postprandial time was set at 1.5 h (*n* = 3), 3 h (*n* = 3), and 6 h (*n* = 3), and the subjects ingested identical meals. After collecting plasma and CSF, we performed a global metabolomics analysis to provide an overview of the postprandial alterations in CSF and plasma metabolites. The present study provides fundamental information about postprandial alteration in CSF metabolites and facilitates the application of CSF for biomarker screening using metabolomics.

## 2. Results

### 2.1. Postprandial Effects on Global Profiles of CSF Metabolites

**Figure 1:**
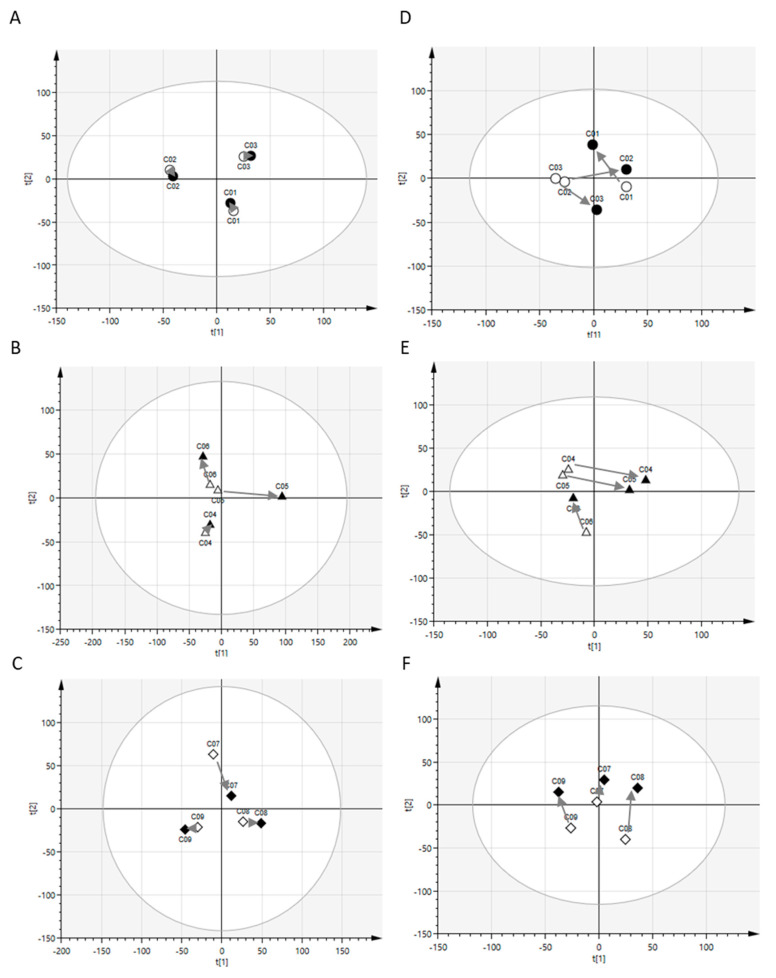
Principal component analysis (PCA) model of hydrophilic metabolites. Data obtained for cerebrospinal fluid (CSF) (**A**–**C**) and plasma (**D**–**F**) from preprandial (white) and postprandial (black) subjects were analyzed. The postprandial periods were 1.5 h (circle), 3 h (triangle), and 6 h (diamond). The grey arrows indicate the change in levels of different metabolites in preprandial and postprandial samples of individual subjects. The circle around the samples indicates the tolerance ellipse determined by Hotelling’s T^2^ with a two-dimensional score plot (95% tolerance was set for all the data analyses in this study). Data points outside the ellipse represent the outliers (none in this study).

Because hydrophilic metabolites, such as amino acids and sugars, and hydrophobic metabolites, namely, lipids, are transported across the cell membrane via different mechanisms, we examined the hydrophilic metabolites and lipids separately. We detected 150 and 130 hydrophilic metabolites in the CSF and plasma, respectively (Supplementary Tables S1 and S2). These metabolite levels were subjected to principal component analysis (PCA) to visualize the postprandial effects on global profiles of hydrophilic metabolites in CSF and plasma. As shown in Figure 1A–C, individual plots of postprandial samples of CSF hydrophilic metabolites were positioned similar to the corresponding plots of preprandial samples at all the time points studied. On the contrary, plots of plasma hydrophilic metabolites tended to cluster into preprandial and postprandial samples (Figure 1D–F).

**Figure 2:**
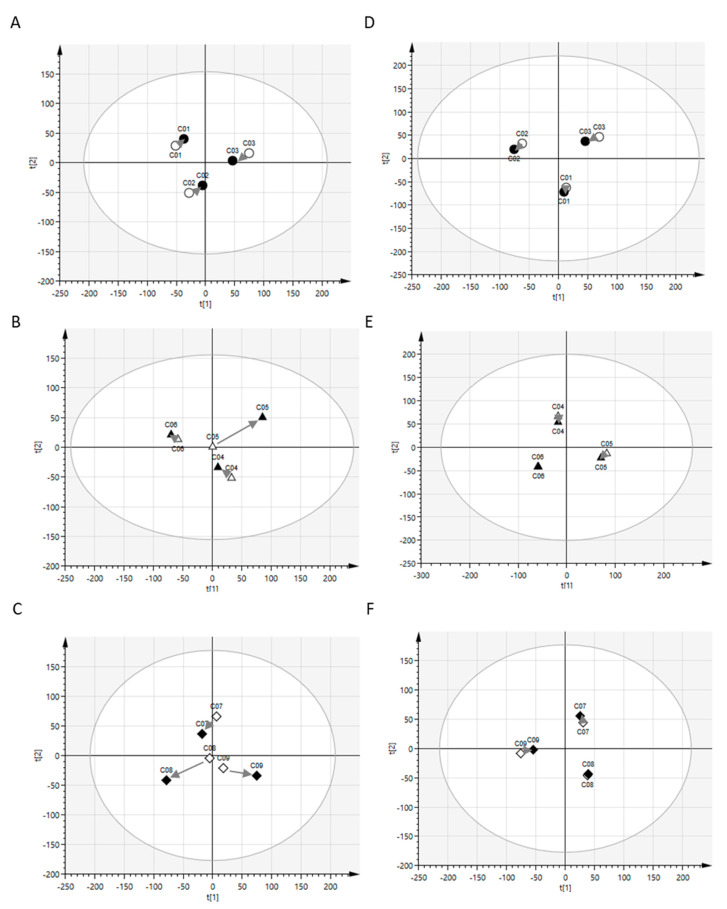
PCA model of lipids. Data obtained from the CSF (**A**–**C**) and plasma (**E**–**F**) of preprandial (white) and postprandial (black) subjects were analyzed. The prandial periods were 1.5 h (circle), 3 h (triangle), and 6 h (diamond). The grey arrows indicate the change in levels of different metabolites in preprandial and postprandial samples of individual subjects. The circle around the samples indicates the tolerance ellipse determined by Hotelling’s T^2^ with a two-dimensional score plot (95% tolerance was set for all the data analyses in this study). Data points outside the ellipse represent the outliers (none in this study).

Next, lipid levels were subjected to PCA to visualize the postprandial effects on global profiles of CSF and plasma lipids. In the present study, 263 and 340 lipids were detected in CSF and plasma, respectively (Supplementary Tables S3 and S4). As shown in Figure 2A–C, individual plots of postprandial samples of CSF lipids were positioned similar to the corresponding plots of preprandial samples at all time points. Further, plots of plasma lipids showed similar positions for preprandial and postprandial samples from the same donor (Figure 2D–F). Overall, these results indicate that postprandial effects on the global profiles of CSF hydrophilic metabolites and lipids were limited and weaker than those of interindividual variations. The postprandial effects on the global profiles of plasma lipids were also limited, but those on the global profiles of plasma hydrophilic metabolites were evident compared to those of interindividual variations.

### 2.2. Postprandial Effects on Individual CSF Metabolites

**Figure 3:**
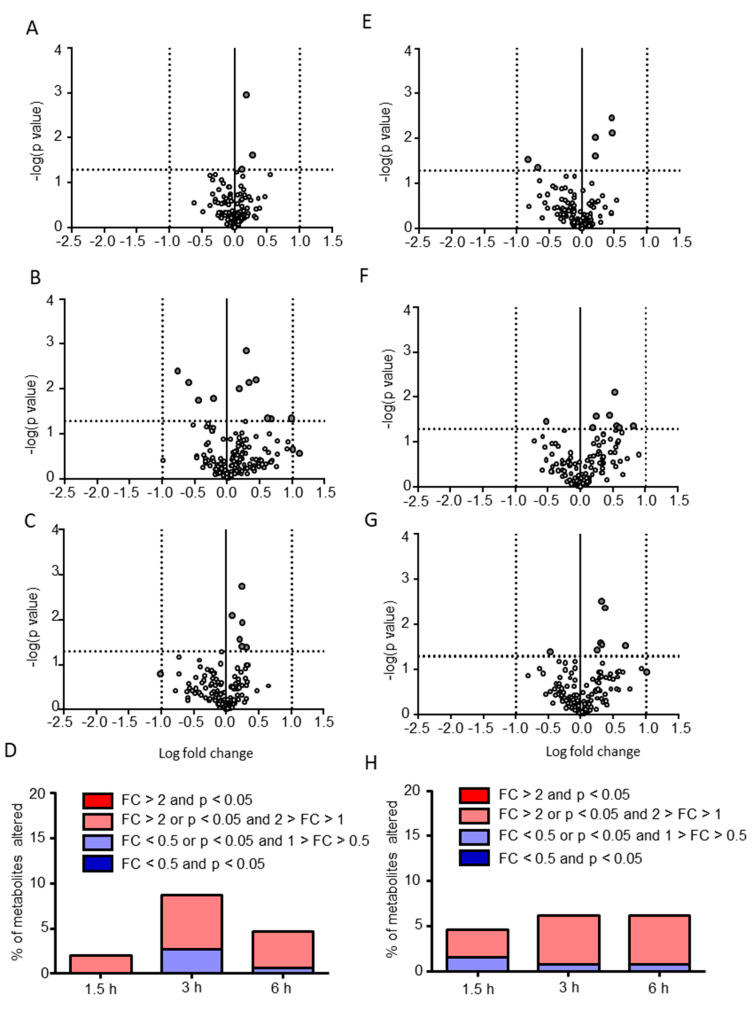
Putative hydrophilic metabolites altered by postprandial effects. Volcano plots (**A**–**C**,**E**–**G**) comparing the hydrophilic metabolites obtained from preprandial and postprandial subjects are shown. The postprandial periods were 1.5 h (**A**,**E**), 3 h (**B**,**F**), and 6 h (**C**,**G**) and the sample sources were CSF (**A**–**C**) and plasma (**E**–**G**). The *p*-values and fold changes (FCs) are plotted as −log10 and log2 values, respectively. Each dot indicates each hydrophilic metabolite. The threshold values (*p* < 0.05 or fold change > 2) are indicated by dotted lines. The percentage of putative hydrophilic metabolites in CSF (**D**) and plasma (**H**) are also demonstrated.

After characterizing the postprandial effects on the global profiles of CSF metabolites, we analyzed the differences in individual metabolite levels between preprandial and postprandial samples. For putative metabolites altered by the postprandial effects, either over 2-fold changes or statistically significant differences between the preprandial and postprandial samples were determined. As shown in Figure 3A–D, only 3, 13, and 7 (2%, 8.7%, and 4.7% of the total molecules) of the CSF hydrophilic metabolites were determined as putative metabolites altered by postprandial effects at 1.5, 3, and 6 h, respectively, and no CSF hydrophilic metabolite showed both over 2-fold change and statistical significance (Table 1). There were no common putative hydrophilic metabolites among the time points. We found that 5-aminovaleric acid, acetoacetic acid, and N-acetyltyrosine ethyl ester were putatively increased postprandially at 1.5 h. N-acetylneuraminic acid, mevalonic acid, cystine, N-formylmethionine, 2-hydroxyglutaric acid, dimethylglycine, glucose, nicotinamide, and phosphoric acid were also increased putatively, whereas arginine, creatine, 4-hydroxybenzoic acid, and p-hydroxyphenylpyruvic acid were putatively decreased postprandially at 3 h. Further, 3-hydroxy-2-methylbutanoic acid, fucose, glucaric acid, glucuronic acid, inositol, and tyramine were putatively increased, whereas citramalic acid was putatively decreased postprandially at 6 h. These results indicate that the postprandial effects on individual CSF hydrophilic metabolites were limited. Moreover, only 6, 8, and 8 (4.6%, 6.1%, and 6.1% of the total molecules) plasma hydrophilic metabolites were determined postprandially at 1.5, 3, and 6 h, respectively, and no plasma hydrophilic metabolite showed both over 2-fold change and statistical significance (Figure 3E–H and Supplementary Table S5). Only dCMP was commonly extracted as a putative hydrophilic metabolite among the 3 and 6 h postprandial time points.

**Figure 4:**
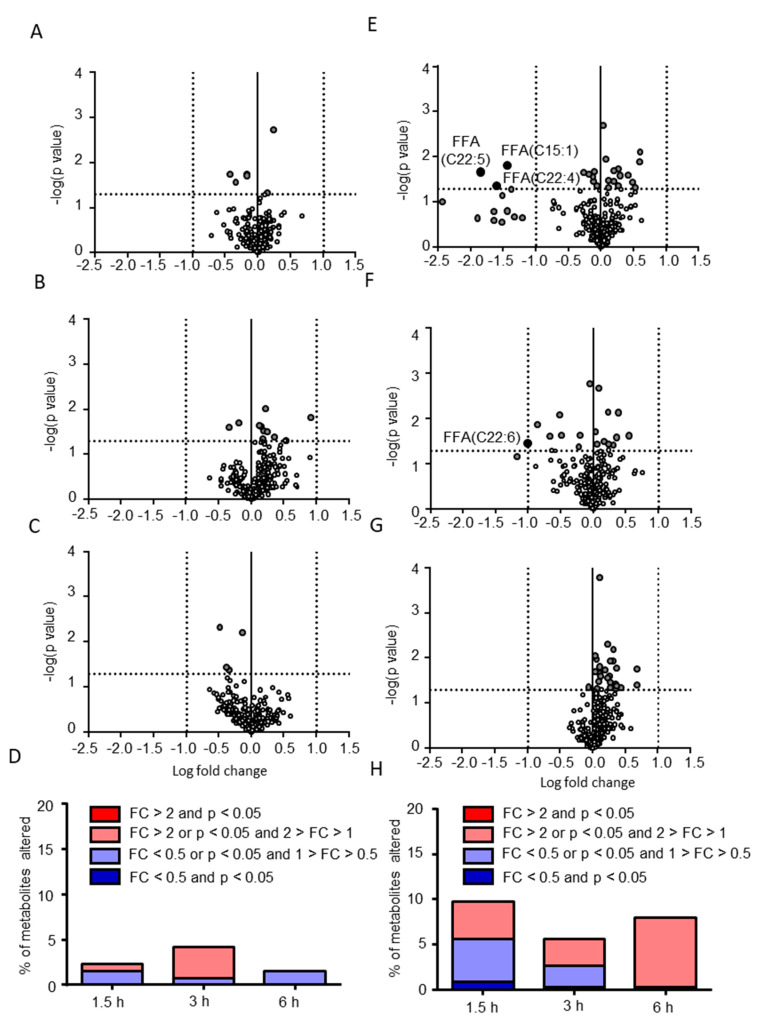
Putative lipids altered by the postprandial effects. Volcano plots (**A**–**C**,**E**–**G**) comparing the lipids obtained from preprandial and postprandial subjects are shown. The postprandial periods were 1.5 h (**A**,**E**), 3 h (**B**,**F**), and 6 h (**C**,**G**) and sample sources were CSF (**A**–**C**) and plasma (**E**–**G**). The *p*-values and fold changes (FCs) were plotted as −log10 and log2 values, respectively. Each dot indicates each lipid. The threshold values (*p* < 0.05 or fold change > 2) are presented as dotted lines. The percentage of putative lipids in the CSF (**D**) and plasma (**H**) are also demonstrated. FFA: free fatty acid.

Next, putative lipid metabolites altered by the postprandial effects were determined. As shown in Figure 4A–D, 6, 11, and 4 (2.3%, 4.2%, and 1.5% of the total molecules) CSF lipids were determined as putative metabolites altered by the postprandial effects at 1.5, 3, and 6 h, respectively, and no CSF lipid showed both over 2-fold change and statistical significance (Table 2). There was no common putative hydrophilic metabolite among the time points. Phosphatidylethanolamine (PE)(aa-38:4), and SM(23:2) were putatively increased, whereas free fatty acids (FFA)(C14:0), FFA(C20:0), FFA(C22:0), and PE(aa-34:1) were putatively decreased postprandially at 1.5 h. Ceramide (Cer)(18:0), cholesterolester (CE)(a-22:6), FFA(C18:1), lysophosphatidylethanolamine (LPE)(a-18:0), phosphatidylcholine (PC)(aa-40:5), PC(aa-42:7), PC(ae-38:5), PC(ae-38:6), and PE(ae-36:5) were putatively increased, whereas LPE(a-18:1) and Sulfatide(Hex/20:0) were putatively decreased postprandially at 3 h. FFA(C14:1), PE(ae-34:2), PE(ae-36:4), and Sulfatide(Hex/16:0) were putatively decreased postprandially at 6 h. These results indicate that the postprandial effects on individual CSF lipids were very limited. On the contrary, 3 and 1 plasma lipids showed statistically significant differences with over 2-fold change at 1.5 and 3 h (Figure 4E–H and Supplementary Table S6). In addition, 33, 19, and 27 (9.7%, 5.6%, and 7.9% of the total molecules) plasma lipids were determined as putative metabolites altered by the postprandial effects at 1.5, 3, and 6 h, respectively. Moreover, 6 FFAs were commonly extracted as putative hydrophilic metabolites among the 1.5 and 3 h postprandial samples. The plasma lipids that showed statistically significant differences with over 2-fold change at 1.5 and 3 h were all FFAs and were decreased in the postprandial state (Supplementary Figure S1). This decrease was conserved up to 3 h and was diminished at 6 h. This decreased level of FFAs in plasma was in agreement with the results from previous studies [15,23] and validate that metabolites sensitive to the postprandial effect were evaluable in the present study even in the small sample size.

### 2.3. Classification of Putative CSF Metabolites Altered by Postprandial Effects

**Figure 5:**
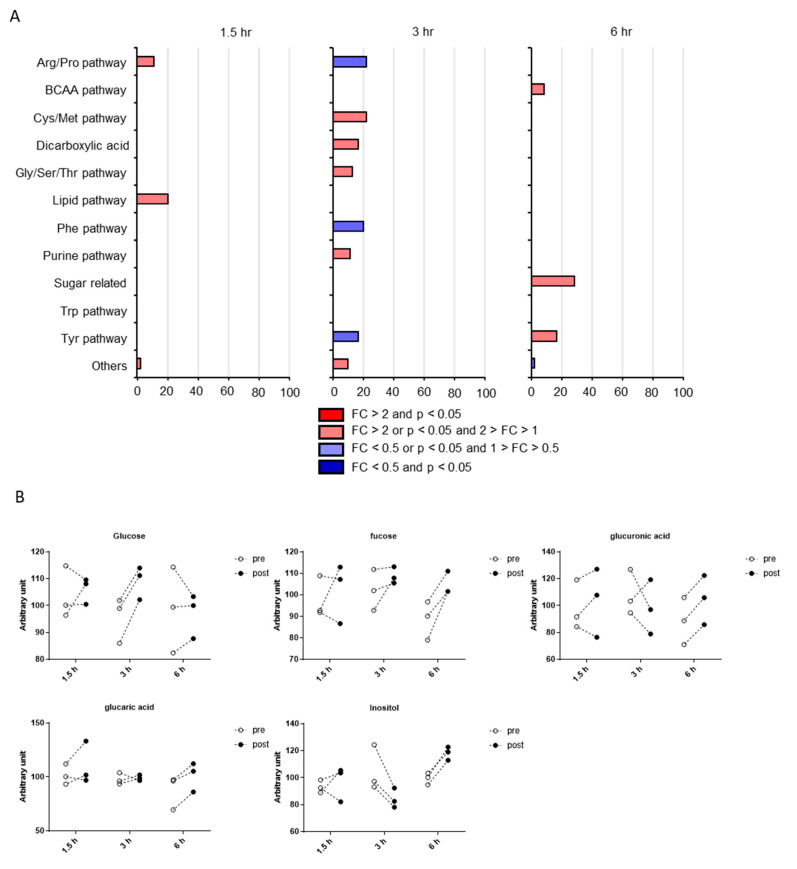
Pathway occupancy rates of putative hydrophilic metabolites altered by postprandial effects. (**A**) Pathway occupancy rates are listed as percentages. Statistical significance using hypergeometric tests is indicated as follows: * *p* < 0.05, ** *p* < 0.01. (**B**) Levels of hydrophilic metabolites in the sugar-related pathway and their precursor, glucose.

Although our results demonstrate that the postprandial effects on CSF metabolites were limited, the classification of putative CSF metabolites altered by postprandial effects might be important for future biomarker research. As shown in Figure 5A, the putative CSF hydrophilic metabolites were distributed among various pathways at all time points and no common pathway was enriched among all time points. The most enriched pathway was sugar related and included 28.6% of the determined metabolites at 6 h. In addition, the hypergeometric tests revealed the statistical significance of the sugar-related pathway at 6 h. Individual plots of putative CSF metabolites altered by postprandial effects in the sugar-related pathway are demonstrated in Figure 5B. Along with metabolites in the sugar-related pathway, their precursor, glucose, was altered at 3 h in CSF.

**Figure 6:**
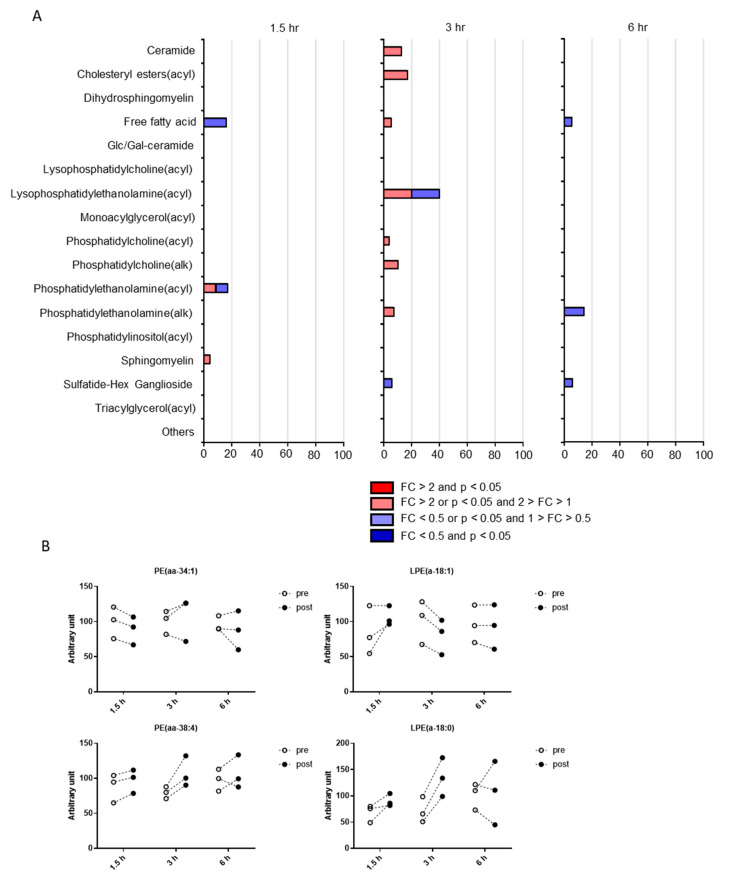
Pathway occupancy rates of putative lipids altered by postprandial effects. (**A**) Pathway occupancy rates are listed as percentages. Statistical significance using hypergeometric tests is indicated as follows: * *p* < 0.05, ** *p* < 0.01. (**B**) Levels of lipids in the lysophosphatidylethanolamine (LPE) pathway and their precursor, phosphatidylethanolamine.

On the contrary, putative CSF lipids were also distributed among various pathways at all time points and no common pathway was enriched among all the time points (Figure 6A). Statistical significance using hypergeometric tests was observed in the pathways of free fatty acids (1.5 h), phosphatidylethanolamine (acyl; 1.5 h), lysophosphatidylethanolamine (3 h), and phosphatidylethanolamine (alk; 6 h). The most enriched pathway was lysophosphatidylethanolamine (LPE), which included 40% of the determined metabolites at 3 h. The individual plots of putative CSF metabolites altered by postprandial effects in the LPE pathway are demonstrated in Figure 6B. Along with the metabolites in the LPE pathway, their precursor lipid, phosphatidylethanolamine (PE), was altered at 1.5 h in CSF.

## 3. Discussion

In the present study, we examined the postprandial effects on the global profiles of CSF metabolites. No clear alteration in the global profiles of CSF metabolites was observed at any time point of the postprandial state. Further, the postprandial effects had a far lower impact compared with interindividual variations. In addition, only a limited number of metabolites were determined to be putatively altered by the postprandial effect and this was not conserved at any time point. The observed impact of the postprandial effects on CSF metabolites was lower than that on plasma metabolites, suggesting that the global profiles of CSF metabolites are not directly associated with food intake. Moreover, we observed a reduction in FFA levels in four plasma lipids, which was in agreement with the results from previous studies [15,23]. Although the low statistical power caused by the limited sample size remains a concern, the findings of the present study supported by earlier findings [15,23] partially validate that the metabolites sensitive to the postprandial effect were evaluable in the present study.

Although none of the CSF metabolites showed both statistical significance and over 2-fold changes at any time point, several metabolites were characterized as putatively altered metabolites by postprandial effects. These metabolites have also been characterized as metabolites altered in brain-related diseases in previous studies applying CSF metabolomics. For example, decreased levels of CSF inositol were characterized in patients with the early stage of multiple sclerosis [24]. In addition, cysteine has been identified as one of the 27 CSF metabolites distinguishable in different types of brain tumors [25]. Glucose levels in CSF are also demonstrated to be decreased in patients with Alzheimer’s disease [26]. However, these studies have rarely described control of the postprandial state. Therefore, when applying these metabolites as biomarkers for brain-related diseases, the postprandial effects should be considered.

The metabolites characterized as putative metabolites altered by postprandial effects are classified into several pathways such as sugar-related and LPE pathways. Metabolites in the sugar-related pathway were products of glucose metabolism, such as glucuronic acid and inositol, and were increased in CSF postprandially at 6 h. However, their precursor, glucose, was increased in CSF postprandially at 3 h and this increase was diminished at 6 h. This observation of increased glucose in CSF postprandially at 3 h was in agreement with a previous report [27]. Taken together, increased levels of metabolites in the sugar-related pathway postprandially at 6 h were a consequence of glucose metabolism in brain tissues. Unlike in CSF, no putative alteration of glucose was observed in plasma samples in the present study. However, it is well known that blood glucose levels peak at 0.5 h after food intake and return to the baseline at 1–2 h [27,28]. The possibility remains that postprandial effects might have occurred within a shorter time frame than 1.5 h. 

LPEs are lysophospholipids of phosphatidylethanolamine, which is one of the two major phospholipids in the cellular membrane [29,30]. In the present study, one LPE(a-18:0) was increased, whereas another LPE(a-18:1) was decreased postprandially at 3 h. Moreover, a predictive precursor of LPE(a-18:0), PE(aa-38:4), by the loss of fatty acid (20:4), was increased, whereas a predictive precursor of LPE(a-18:1), PE(aa-34:1), by the loss of fatty acid (16:0), was decreased postprandially at 1.5 h. Therefore, postprandial effects alter the levels of several PEs and subsequently alter the levels of several LPEs in CSF. Because the postprandial remodeling of plasma phospholipids has been reported [31,32], even limited postprandial effects could result in the remodeling of CSF phsopholipids. So far, the mechanism underlying CSF phospholipid remodeling remains unclear. However, the impact of postprandial remodeling of plasma phospholipids is reported to be related to the diet composition and relative size of the endogenous phospholipid pools. Therefore, diet composition and phospholipids pooled in CSF should be also considered when CSF lipids are used as biomarkers.

There were several limitations in the present study. First, this study was performed with a limited number of subjects. Although we recruited only male participants in a limited age range and served them identical meals, the presence of false positives and false negatives cannot be denied. Moreover, the low statistical power of the small sample size, leading to false negatives, cannot be overruled. Second, we only examined the postprandial effects of one specific meal. Previous reports have demonstrated that phospholipid remodeling in plasma is dependent on the source of fats [31,32] and could apply to the CSF phospholipids as well. Third, although we collected self-reported healthy subjects without any medication for at least 1 week, unawareness of disease is inevitable. Fourth, in the present study, we did not control the subjects’ alcohol and food habits that might have affected the postprandial responses of metabolites. Alcohol intake, as well as a high fat diet, has been well demonstrated to cause alterations of expressions of various hepatic genes, including genes related to energy homeostasis and diet metabolism [33,34,35], and that might result in an altered postprandial response. Last, lumbar puncture is an invasive approach and has been reported to increase the levels of several hormones in plasma [36]. Its effect on the alteration of different metabolites in plasma and CSF postprandial samples in the present study cannot be denied. Therefore, to overcome these limitations and to determine the general role of postprandial effects in CSF metabolite levels, a large-scale study with controlling factors affecting the postprandial responses is required in future.

In conclusion, the present study suggests that the global levels of CSF metabolites are not directly associated with food intake at least during the postprandial time of 1.5–6 h. It further indicates the possibility that except for several putatively altered CSF metabolites, postprandial effects are not a major concern when applying CSF metabolomics for screening biomarkers. This pilot study provides valuable fundamental information for biomarker research using CSF metabolomics and would be useful for a large-scale CSF metabolomics study.

## 4. Materials and Methods

### 4.1. Subjects and Sample Collection

Subjects were recruited at the National Center of Neurology and Psychiatry (NCNP), Tokyo, Japan, through an announcement in a local free magazine or on our website. All participants were nonsmoking, self-reportedly healthy male volunteers (age: 30–44; body mass index: 18.9–25.5) without any medications for at least 1 week. Subjects were excluded if they had a history of central nervous system disease, severe head injury, or substance abuse. Heavy exercise causing muscle pain was prohibited from 3 days before the test. The last meal (with no alcohol) was taken before 9:00 PM on the day before the test, and only water was allowed afterward.

At 9:30 AM of the test day, subjects received the first lumbar puncture. At 10:00 AM, they began eating the same set meal including a hamburger steak, rice, fries, green salad, and miso soup (total: ~880 kcal). They ingested the entire meal within 30 min and only water intake was allowed afterwards. The second lumbar punctures were performed at 1.5, 3, or 6 h after starting the meal (3 cases per condition). Between the meal and the second lumbar puncture, they could stay in the room or walk freely inside the NCNP with a monitoring psychologist but could not lie on the bed. 

CSF samples were obtained by lumbar puncture as described previously [37]. After neurologic examination, each participant received local anesthesia followed by a lumbar puncture at the L3-4 or L4-5 using an atraumatic pencil point needle (Uniever 22G, 75 mm, Unisis Corp., Tokyo, Japan). The initial 2 mL of CSF was collected for laboratory tests, including cell count, total protein, and glucose. Thereafter, 8–10 mL of CSF was collected in a low-protein adsorption tube (PROTEOSAVE SS 15 mL Conical tube, Sumitomo Bakelite Co., Japan) and immediately chilled on ice. The CSF was centrifuged (4000× *g*, 10 min, 4 °C) and the supernatant was dispensed into 0.5 mL aliquots in low-protein adsorption tubes (PROTEOSAVE SS 1.5 mL Slim tube, Sumitomo Bakelite Co.) and stored in a deep freezer (−80 °C) before use. 

Simultaneously, blood samples were collected by venipuncture into 7 ml EDTA-2Na-containing vacuum blood collection tubes (VENOJECT II, TERUMO, Tokyo, Japan). The samples were immediately centrifuged (2500× *g*, 10 min, 4 °C), dispensed in screw-capped polypropylene tubes (96 Jacket Tubes 1.3 ml internal type, FCR&Bio Co., Ltd., Japan), and stored in a deep freezer (−80 °C) before use.

This study was conducted in accordance with the Declaration of Helsinki and was approved by the ethics committee of the National Center of Neurology and Psychiatry (No. A2019-088). Written informed consent was obtained from all the participants.

### 4.2. Metabolomic Analysis

The metabolite profiles of samples were obtained using three analytical methods: gas chromatography/tandem mass spectrometry (GC/MS/MS), hydrophilic interaction liquid chromatography/tandem mass spectrometry (HILIC/MS/MS), and lipidomic analysis.

For GC/MS/MS analysis, plasma and CSF samples were extracted with 9 volumes of methanol and were centrifuged at 21,500× *g* for 5 min. The supernatant was homogenized with internal standards labeled using stable isotopes and was dried under a stream of nitrogen gas. The dried samples were derivatized by oximation and trimethylsilylation. The derivatized analytes were injected into an Agilent 7890A series gas chromatography system (Agilent Technologies Inc., CA, USA). Chromatographic separation was performed in a J&W Scientific DB-5MS-DG column (30 m × 0.25 mm i.d., df = 0.25 μm, Agilent Technologies Inc.) using a temperature gradient, which increased at 10 °C/min from 60 to 325 °C, with helium gas flow at a rate of 1 mL/min. The eluted metabolites were introduced into an Agilent 7010B triple-quadrupole mass spectrometer (Agilent Technologies Inc.) for electron impact ionization and were scanned in the multiple reaction monitoring (MRM) mode. The MRM peak area was calculated using MassHunter (Agilent Technologies Inc.).

For HILIC/MS/MS analysis, a sample was mixed with 9 volumes of methanol and was centrifuged at 21,500× *g* for 5 min. Next, 100 μL of the supernatant was mixed with 25 μL of 100 mM ammonium formate. The mixture was then vortexed and centrifuged at 21,500× *g* for 5 min. The supernatant was applied to the LC/MS/MS system comprising a UHPLC Nexera liquid chromatography system (Shimadzu Co., Kyoto, Japan) and a 5500QTRAP mass spectrometer (AB Sciex pte. Ltd., Toronto, Canada). The analytes were separated through a ZIC-cHILIC column (2.1 × 100 mm, 3 μm, Merck Millipore, Darmstadt, Germany) at a temperature of 30 °C with a gradient elution of the mobile phases A (10 mM ammonium formate aqueous solution) and B (acetonitrile) at a flow rate at 0.4 mL/min. The gradient program was as follows: 0–1.5 min, 97% B; 1.5–5 min, 97–75% B; 5–7 min, 75% B; 7–10 min, 75–40% B; 10–12 min 40% B; 12–13 min, 40–10% B; 13–16 min, 10% B; and 16–25 min, 97% B. The eluent was ionized using electrospray ionization and was scanned in the MRM mode, as reported by Yuan et al. [38]. MRM data were processed using MultiQuant 3.0 (ABSciex, Pvt. Ltd.).

The method of lipidomic analysis has been previously reported by Satomi et al. [39]. A sample was extracted with 9 volumes of ethanol and centrifuged at 21,500× *g* for 5 min. The supernatant was injected into a CORTECS T3 column (2.1 × 50 mm, 2.7 μm, 120Å, Waters Co., Milford, MA, USA) maintained at a temperature of 60 °C, and the lipids were separated by gradient elution of the mobile phases A (MilliQ water with 0.01% acetic acid, 1 mM NH_3_, and 10 μM EDTA-2Na) and B (0.001% acetic acid and 0.2 mM NH_3_ in ethanol/isopropanol (1:1)), with the flow rate set to 0.7 mL/min. The gradient elution condition was as follows: 0–1 min 0% B; 1–13 min 0–100% B; 13–15 min 100% B; and 15–18 min 0% B. The eluents were introduced into an Orbitrap XL Mass Spectrometer (Thermo Fisher Scientific, Inc., San Jose, CA, USA). The mass spectra were acquired in a data-dependent mode. Precursor ion spectra were scanned using the orbitrap analyzer at 60,000 full width at half maximum resolution and 400 *m*/*z*, and the product ion spectra by higher energy collisional dissociation were in the linear ion trap. The raw LC/MS data were processed using Expressionist Refiner MS software (ver. 8.2, Genedata AG, Basel, Switzerland). Each MS peak was compared with the in-house lipid database containing information on retention time, exact mass, and the preferred adduct ion species, and its structure was estimated.

The identified hydrophilic metabolites from CSF and plasma samples were assigned the HMDB ID, Pubchem CID, and formula, whereas the identified lipids were assigned the formulas.

### 4.3. Data Cleaning and Statistical Analyses

All the obtained metabolite data were median scaled prior to statistical analysis and the median was set as 100. In addition, due to the small sample size, metabolites with zero values in any sample and with high interindividual variations (median value > 120 or <80, or coefficient of variation > 50% in the preprandial state) were removed in the present study. The putative metabolites showing alteration were confirmed either by a significance test through paired *t* tests (*p* < 0.05 was considered significant) or by a fold-change of >2 folds or <0.5 fold.

### 4.4. Principal Component Analysis (PCA)

Processed metabolite data were loaded into SIMCA-P+ 14 (Umetrics, Umea, Sweden), pareto scaled, and were analyzed using PCA-X to visualize variance among the groups evaluated in this study. The PCA-X results were provided as score plots to represent the similarity in the overall metabolic profiles. The tolerance ellipse was drawn by Hotelling’s T^2^ with a two-dimensional score plot by setting the tolerance at 95%. 

### 4.5. Pathway Occupancy Analysis

To construct pathway occupancy maps, pathways represented by more than five metabolites were selected and those represented by less than five metabolites were merged. Each pathway was scored with statistical significance and fold changes of metabolites within the pathway (*p* < 0.05, scored as 1; *p* > 0.05, scored as 0; fold change > 2 or <0.5, scored as 1; 0.5 < fold change < 2, scored as 0). The scored values were divided by the number of metabolites within specific pathways, and the resulting ratios indicated the metabolites that showed statistically significant results within a pathway. The statistical significance of each pathway occupation was determined by hypergeometric tests.

## Figures and Tables

**Table 1:** Putative CSF hydrophilic metabolites altered by postprandial effects.

Group	Name	*p*-Value			Fold Change		
		1.5 h	3 h	6 h	1.5 h	3 h	6 h
Amino sugar	N-Acetylneuraminic acid	0.53	**<0.01**	0.48	0.9	1.36	1.18
Arg/Pro pathway	5-Aminovaleric acid	**0.02**	0.14	0.64	1.22	1.71	0.83
Arg/Pro pathway	Arginine	0.97	**<0.01**	0.75	1.01	0.59	1.08
Arg/Pro pathway	Creatine	0.66	**0.02**	0.95	1.04	0.73	1.01
BCAA pathway	3-Hydroxy-2-methylbutanoic acid	0.61	0.07	**0.04**	1.1	0.84	1.24
C5-Branched dibasic acid pathway	Citramalic acid	0.28	0.4	0.16	0.65	1.39	**0.49**
Cholesterol pathway	Mevalonic acid	0.93	**<0.01**	0.69	0.99	1.27	0.94
Cys/Met pathway	Cystine	0.54	0.22	0.61	1.05	**2.01**	1.08
Cys/Met pathway	N-Formylmethionine	0.72	**<0.01**	0.98	1.06	1.23	1
Dicarboxylic acid	2-Hydroxyglutaric acid	0.14	**0.05**	0.38	1.1	1.54	0.7
Gly/Ser/Thr pathway	Dimethylglycine	0.47	0.27	0.75	1.03	**2.16**	0.92
Glycolysis	Glucose	0.69	**<0.01**	0.76	1.02	1.14	0.98
Lipid pathway	Acetoacetic acid	**0.05**	0.57	0.72	1.09	1.21	1.02
N-acetyl-amino acid	N-Acetyltyrosine ethyl ester	**<0.01**	0.77	0.26	1.14	0.91	0.77
Nicotinate and nicotinamide pathway	Nicotinamide	0.22	**0.05**	0.55	1.29	1.99	0.8
Phe pathway	4-Hydroxybenzoic acid	0.53	**<0.01**	0.76	0.84	0.67	1.07
Purine pathway	Phosphoric acid	0.94	**0.05**	0.13	0.98	1.6	0.82
Sugar related	Fucose	0.63	0.26	**0.04**	1.05	1.06	1.18
Sugar related	Glucaric acid	0.34	0.8	**0.03**	1.09	1.01	1.15
Sugar related	Glucuronic acid	0.53	0.54	**<0.01**	1.05	0.91	1.18
Sugar related	Inositol	0.68	0.07	**0.01**	1.04	0.8	1.19
Tyr pathway	p-Hydroxyphenylpyruvic acid	0.3	**0.02**	0.6	0.87	0.87	1.14
Tyr pathway	Tyramine	0.87	0.34	**<0.01**	0.99	1.06	1.07

Underlined values in bold cases reached the threshold p value or fold change. Fold changes were calculated by dividing the average scaled values of postprandial samples in each group with those of pre-prandial samples.

**Table 2:** Putative CSF lipids altered by the postprandial effects.

Group	Name	*p*-Value			Fold Change		
		1.5 h	3 h	6 h	1.5 h	3 h	6 h
Ceramide	Cer(18:0)	0.13	**0.02**	0.66	1.09	1.11	1.01
Cholesteryl esters(acyl)	CE(a-22:6)	0.97	**0.02**	0.27	1.01	1.09	0.80
Free fatty acid	FFA(C14:0)	**0.02**	0.56	0.75	0.89	1.14	1.05
Free fatty acid	FFA(C14:1)	0.88	0.82	**<0.01**	1.06	1.03	0.72
Free fatty acid	FFA(C18:1)	0.13	**0.05**	0.41	0.65	1.45	1.20
Free fatty acid	FFA(C20:0)	**0.03**	0.12	0.43	0.79	1.48	1.29
Free fatty acid	FFA(C22:0)	**0.02**	0.11	0.59	0.75	1.23	1.25
Lysophosphatidylethanolamine(acyl)	LPE(a-18:0)	0.13	**0.02**	0.85	1.33	1.89	1.05
Lysophosphatidylethanolamine(acyl)	LPE(a-18:1)	0.25	**0.03**	0.46	1.26	0.79	0.97
Phosphatidylcholine(acyl)	PC(aa-40:5)	0.91	**0.03**	0.84	1.00	1.19	0.98
Phosphatidylcholine(acyl)	PC(aa-42:7)	0.27	**0.05**	0.25	1.03	1.13	1.08
Phosphatidylcholine(alk)	PC(ae-38:5)	0.66	**<0.01**	0.47	1.01	1.16	0.90
Phosphatidylcholine(alk)	PC(ae-38:6)	0.42	**0.03**	0.58	0.98	1.14	0.94
Phosphatidylethanolamine(acyl)	PE(aa-34:1)	**0.02**	0.50	0.54	0.89	1.08	0.91
Phosphatidylethanolamine(acyl)	PE(aa-38:4)	**0.05**	0.08	0.49	1.11	1.35	1.09
Phosphatidylethanolamine(alk)	PE(ae-34:2)	0.39	0.33	**<0.01**	1.06	1.15	0.91
Phosphatidylethanolamine(alk)	PE(ae-36:4)	0.63	0.43	**0.04**	1.05	1.11	0.77
Phosphatidylethanolamine(alk)	PE(ae-36:5)	0.97	**0.04**	0.09	1.00	1.28	0.85
Sphingomyelin	SM(23:2)	**<0.01**	0.86	0.34	1.18	0.93	0.76
Sulfatide-Hex Ganglioside	Sulfatide(Hex/16:0)	0.56	0.73	**0.04**	0.94	0.95	0.79
Sulfatide-Hex Ganglioside	Sulfatide(Hex/20:0)	0.85	**0.02**	0.20	0.99	0.88	0.84

Underlined values in bold cases reached the threshold p value or fold change. Fold changes were calculated by dividing the average scaled values of postprandial samples in each group with those of pre-prandial samples.

## Supplementary Materials

The following are available online at https://www.mdpi.com/2218-1989/10/5/185/s1, Figure S1: Levels of free fatty acids in plasma, Table S1: Normalized levels of CSF hydrophilic metabolites in the subjects of the present study, Table S2: Normalized levels of plasma hydrophilic metabolites in the subjects of the present study, Table S3: Normalized levels of CSF lipids in the subjects of the present study, Table S4: Normalized levels of plasma lipids in the subjects of the present study, Table S5: Putative plasma hydrophilic metabolites altered by postprandial effects, Table S6: Putative plasma lipids altered by postprandial effects.
