# Supplementary figures and images for: Characterization of Postprandial Effects on CSF Metabolomics: A Pilot Study with Parallel Comparison to Plasma
Source: Metabolites. 2020 May 6;10(5):185. doi: 10.3390/metabo10050185 (PMC7281358; doi:10.3390/metabo10050185)

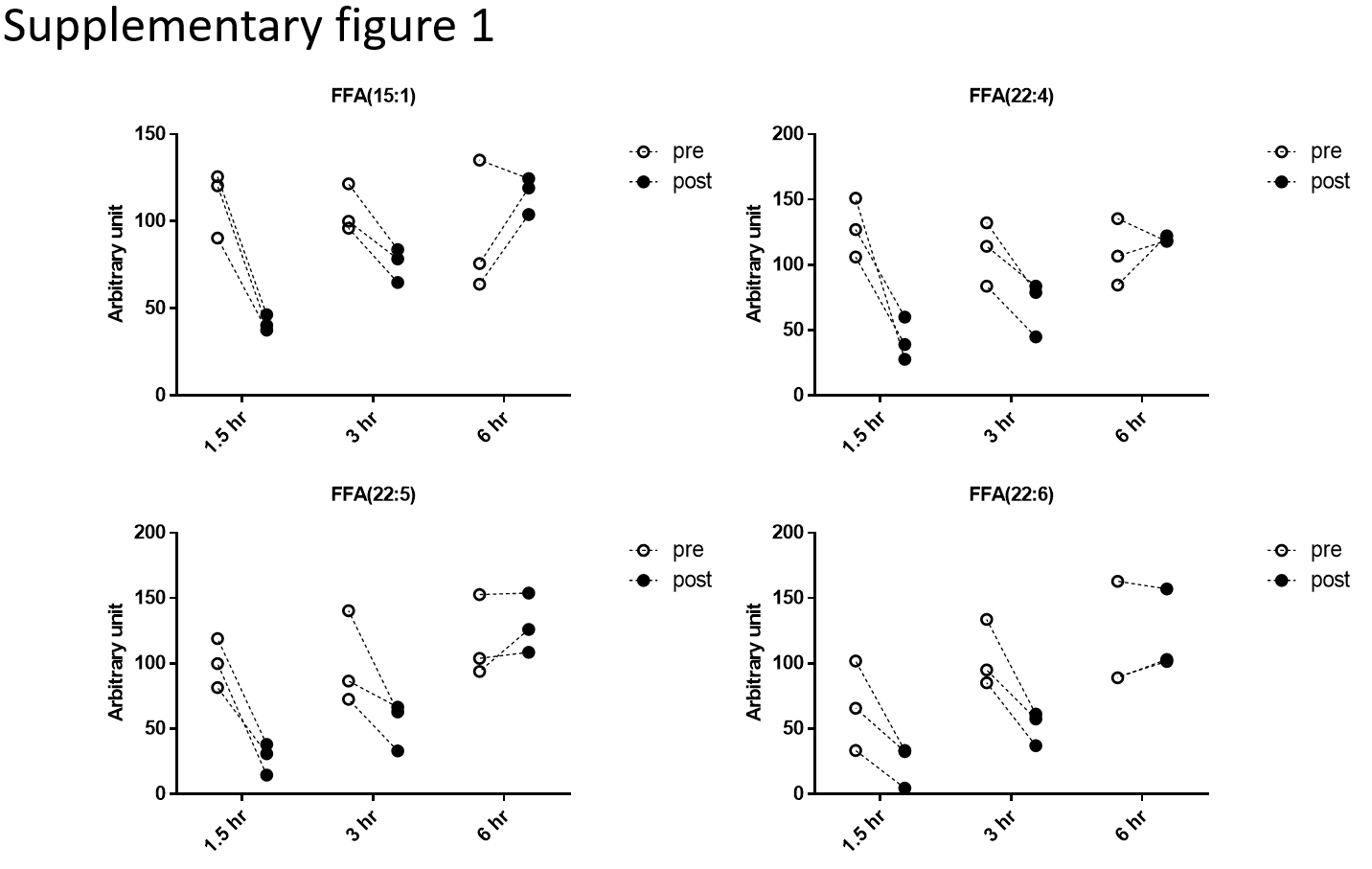

Supplement: Supplementary file 1 [file metabolites-10-00185-s001.zip › Figure S1.jpg]
